# High flow nasal oxygen therapy to avoid invasive mechanical ventilation in SARS-CoV-2 pneumonia: a retrospective study

**DOI:** 10.1186/s13613-021-00825-5

**Published:** 2021-02-27

**Authors:** Nicolas Bonnet, Olivier Martin, Marouane Boubaya, Vincent Levy, Nathan Ebstein, Philippe Karoubi, Yacine Tandjaoui-Lambiotte, Guillaume Van Der Meersch, Johanna Oziel, Marie Soulie, Mohamed Ghalayini, Anais Winchenne, Jean Ralph Zahar, Passem Ahmed, Stéphane Gaudry, Yves Cohen

**Affiliations:** 1 Intensive Care Unit, CHU Avicenne, Groupe Hospitalier Paris Seine Saint-Denis, AP-HP, 125 rue de Stalingrad, 93000 Bobigny, France; 2 Unité de Recherche Clinique, CHU Avicenne, Groupe Hospitalier Paris Seine Saint-Denis, AP-HP, 125 rue de Stalingrad, 93000 Bobigny, France; 3 Unité D’Hygiène Hospitalière, Service de Microbiologie, CHU Avicenne, Groupe Hospitalier Paris Seine Saint-Denis, AP-HP, 125 rue de Stalingrad, 93000 Bobigny, France; 4 Intensive Care Unit, Centre hospitalier de Rambouillet, 5-7 Rue Pierre et Marie Curie, 78120 Rambouillet, France; 5 UFR SMBH, Université Sorbonne Paris Nord, Bobigny, France; 6 Hypoxie Et Poumon, INSERM, U1272 Villetaneuse, France; 7 Common and Rare Kidney Diseases, Sorbonne Université, INSERM, UMR-S 1155 Paris, France; 8 INSERM, U942, 75010 Paris, France

**Keywords:** High flow nasal canula, Acute respiratory failure, COVID-19, Intensive care unit

## Abstract

**Background:**

The efficacy of high flow nasal canula oxygen therapy (HFNO) to prevent invasive mechanical ventilation (IMV) is not well established in severe coronavirus disease 2019 (COVID-19). The aim of this study was to compare the risk of IMV between two strategies of oxygenation (conventional oxygenation and HFNO) in critically ill COVID 19 patients.

**Methods:**

This was a bicenter retrospective study which took place in two intensive care units (ICU) of tertiary hospitals in the Paris region from March 11, to May 3, 2020. We enrolled consecutive patients hospitalized for COVID-19 and acute respiratory failure (ARF) who did not receive IMV at ICU admission. The primary outcome was the rate of IMV after ICU admission. Secondary outcomes were death at day 28 and day 60, length of ICU stay and ventilator-free days at day 28**.** Data from the HFNO group were compared with those from the standard oxygen therapy (SOT) group using weighted propensity score.

**Results:**

Among 138 patients who met the inclusion criteria, 62 (45%) were treated with SOT alone, and 76 (55%) with HFNO. In HFNO group, 39/76 (51%) patients received IMV and 46/62 (74%) in SOT group (OR 0.37 [95% CI, 0.18–0.76] *p* = 0.007). After weighted propensity score, HFNO was still associated with a lower rate of IMV (OR 0.31 [95% CI, 0.14–0.66] *p* = 0.002). Length of ICU stay and mortality at day 28 and day 60 did not significantly differ between HFNO and SOT groups after weighted propensity score. Ventilator-free days at days 28 was higher in HNFO group (21 days vs 10 days, *p* = 0.005). In the HFNO group, predictive factors associated with IMV were SAPS2 score (OR 1.13 [95%CI, 1.06–1.20] *p* = 0.0002) and ROX index > 4.88 (OR 0.23 [95%CI, 0.008–0.64] *p* = 0.006).

**Conclusions:**

High flow nasal canula oxygen for ARF due to COVID-19 is associated with a lower rate of invasive mechanical ventilation.

## Background

Acute respiratory failure (ARF) due to acute hypoxemia is the main manifestation in severe coronavirus disease 2019 (COVID-19). In most severe cases, COVID-19 patients are hospitalized in intensive care unit (ICU) and may require invasive mechanical ventilation (IMV). The need for IMV is associated with high mortality [1, 2].

High flow nasal oxygen (HFNO) is increasingly used for adults hospitalized with ARF. This non-invasive technic delivers warmed, humidified oxygen with a fraction of inspired oxygen (FiO2) up to 1.0 and a maximum flow rate of 60 L/min. In a post hoc subgroup analysis of the Florali study, the use of HFNO reduced the need for IMV in most hypoxemic patients [3]. A recent retrospective study, which deserves to be confirmed, suggested the same benefit in COVID-19 patients [4].

Due to the hypothetic risk of transmission to healthcare workers at the beginning of the sanitary crisis, expert opinion recommend restricting the use of HFNO and limiting the flow rate to 30 L/min for critically ill patients with COVID-19 [5]. This recommendation led intensivists to adopt an early intubation strategy to limit the use of HFNO[6]. However, the risk of bio-aerosol dispersion associated with HFNO has since been questioned[7–9].

Information about clinical outcomes of patients treated by HFNO in ICU for COVID-19 are limited. The aim of this study was to compare the need of IMV between two strategies of oxygenation (conventional oxygenation and HFNO) in critically ill COVID 19 patients.

## Materiel and method

### Study design

This was a bicenter retrospective study which took place in two French hospitals located in Paris area: Hôpital Avicenne, Assistance Publique Hopitaux de Paris and Hôpital de Rambouillet. All adult patients who were diagnosed with COVID-19 according to WHO interim guidance were screened[10], and those with a diagnosis of ARF admitted to the ICUs between March 11, 2020 (i.e., when the first patients were admitted), and May 3, 2020, were included. Acute respiratory failure was defined as respiratory rate of more than 25 per minute and need for standard oxygen ≥ 3L/min to maintain SpO2 ≥ 92%. We did not include patients who were admitted with a decision to withdraw life-sustaining therapy, including do-not-intubate orders, patients who received non-invasive ventilation, and patients who were intubated before ICU admission.

The study was approved by the Medical Ethics Committee of the Hôpital Avicenne with the reference number: CLEA-2020–146.

We followed the statement guidelines of Strengthening the Reporting of Observational Studies in Epidemiology (STROBE) for observational cohort studies [11].

### Oxygenation strategy

All adult patients hospitalized for COVID-19 in participating ICUs required oxygen therapy. Standard-oxygen therapy was applied through a non-rebreather face mask at a flow rate of 6 L/min or more. The oxygen flow rate was adjusted to maintain an oxygen saturation level of more than 92%.

When HFNO was used, oxygen was passed through a heated humidifier (MR850 and AIRVO 2, Fisher and Paykel Healthcare) and applied continuously through large-bore binasal prongs, with a gas flow rate of 60 L per minute and a fraction inspired of oxygen (FiO2) of 1.0 at initiation. The FiO2 in the gas flowing in the system was adjusted to maintain an oxygen saturation level of more than 92%. All patients receiving HFNO wore a surgical mask to prevent SARS-CoV-2 transmission. The HFNO was administered all day long and patients were in supine position until recovery or initiation of IMV.

Before March 27, due to the hypothetic risk of transmission of SARS-CoV-2 to healthcare workers, the use of HFNO was scarce and the flow rate was limited to 30 L per minute according to the French intensive care society guidelines. After March 27, 2020, in the light of a low risk of transmission by bio-aerosolization with HFNO in the literature, we decided to not restrict the use of HFNO and to allow a high flow rate (60 L/min).

Throughout the study period, the decision to intubate was based on clinical characteristics (respiratory rate, worsening of respiratory status, high respiratory-muscle workload) and biological characteristics (arterial partial pressure of oxygen). Worsening respiratory failure was defined by at least two of the following criteria: a respiratory rate of more than 40 breaths per minute; a lack of improvement in signs of high respiratory-muscle workload; the development of copious tracheal secretions; respiratory acidosis with a pH of less than 7.35; and an Spo2 of less than 90% for more than 5 min without technical dysfunction.

At the beginning of the outbreak, training about personal protective equipment dressing and undressing were quickly organized for healthcare workers. All healthcare workers wore an adequate protection including gowns, eye protection (safety glasses or face shield), gloves and masks to protect from droplets, contact or airborne transmission.

### Data collection

Epidemiological, demographic, clinical, laboratory, treatment, and outcome data were extracted from electronic medical records using a standardized data collection form. Laboratory confirmation of SARS-CoV-2 infection was performed by the local health authority.

The data recorded were the following:

#### Epidemiological data

Age, sex, body mass index (BMI), chronic medical histories (chronic cardiac disease, chronic pulmonary disease, diabetes, malignancy),

#### Clinical, biological and radiological characteristics at ICU admission

SAPS II, heart rate, arterial blood pressure, respiratory rate, oxygen flow, time from onset of symptoms to ICU admission, blood count, coagulation profile, serum biochemical tests (including renal and liver function, creatine kinase, lactate dehydrogenase, and electrolytes), myocardial enzymes, interleukin-6 (IL-6), C-reactive protein (CRP), serum ferritin, procalcitonin, arterial blood gas analysis, lactate concentration, chest CT scan.

#### Therapy in ICU

Need for IMV, need for catecholamine infusion, antiviral agents, immunomodulator therapy.

### Outcomes

The primary outcome was the proportion of patients who required IMV after ICU admission.

The secondary outcomes were death 28 days and 60 days after ICU admission, the mean length of stay in ICU and the number of ventilator-free days at day 28. For ventilator-free days, one point was given for each calendar day during the measurement period (i.e., from the first day of admission in ICU to day 28) that a patient was both alive and free of invasive mechanical ventilation, and zero value was given for patients who died before day 28.

We also assessed the number of health care worker contaminations during 2 periods: before March 27, when the use of HFNO was restricted because of the hypothetical risk of aerosol contamination and after March 27, when the use of HFNO was not restricted.

### Statistical analysis

Categorical variables are expressed as number with percentage (%) and continuous variables as mean with standard deviation (SD), or median with interquartile range (IQR). Initial characteristics of the HFNO group and the standard-oxygen therapy group were compared using a Chi-square test or Fisher's exact test for the categorical data, and a t-test or Wilcoxon signed-rank test for continuous data.

The effect of HFNO was assessed using a propensity score analysis to balance the differences in baseline variables between the two groups. The probability for receiving HFNO was calculated by a non-parsimonious logistic regression. Covariates included in this model were selected before analysis: sex, age, BMI, time from onset of symptoms to ICU admission, hypertension, diabetes, and parameters measured at ICU admission: SAPS II, oxygen flow, PaO2, respiratory rate, CRP and chest CT scan severity. Chest CT scan severity was defined by a quantitative evaluation of the abnormal manifestation of chest CT imaging. The abnormal imaging signs included ground glass opacity and consolidation quantified by radiologist. The radiologist estimated the lesion areas on each lung lobe as a percentage of the whole lung lobe [12].

HFNO effect on IMV at 28 days and mortality in ICU at 28 and 60 days were performed with weighted logistic regression using the stabilized inverse probability of treatment weighting (IPTW) [13]. Regarding IMV, there was no competitive risk (no death without IMV at 28 days). Length of ICU stay among patients discharged was compared between oxygenation groups with a weighted log-linear model using the IPTW. The number of ventilation-free days at day 28 was compared using Mann–Whitney U test. Because a non-normal distribution of patients in our sample, we could not perform a weighted logistic regression for this last parameter. Two sensitivity analyses were performed for primary outcome: a truncated IPTW excluding patients with an extreme IPTW (5th-95th percentile) and an analysis excluding patients on HFNO with O2 flow < 50L/min.

To account for missing data, analyses were conducted using multiple imputations by chained equations with 5 imputations obtained after 5 iterations[14]. The number of missing data was low (Additional file 1: Figure S1). The propensity scores came from 10 independent complete data sets and were averaged according to an “across approach” [15]. Covariate balances before and after weighting were assessed by standardized mean differences which came from a complete imputed data set [16].

We also sought to determine predictive factors for IMV for patients receiving HFNO with univariate logistic regressions.

All tests were two-tailed, and the results were considered statistically significant when *p* < 0.05. Analyses were performed using R statistical software version 3.5.2 (R foundation for Statistical Computing, Vienna, Austria).

## Results

**Fig. 1:**
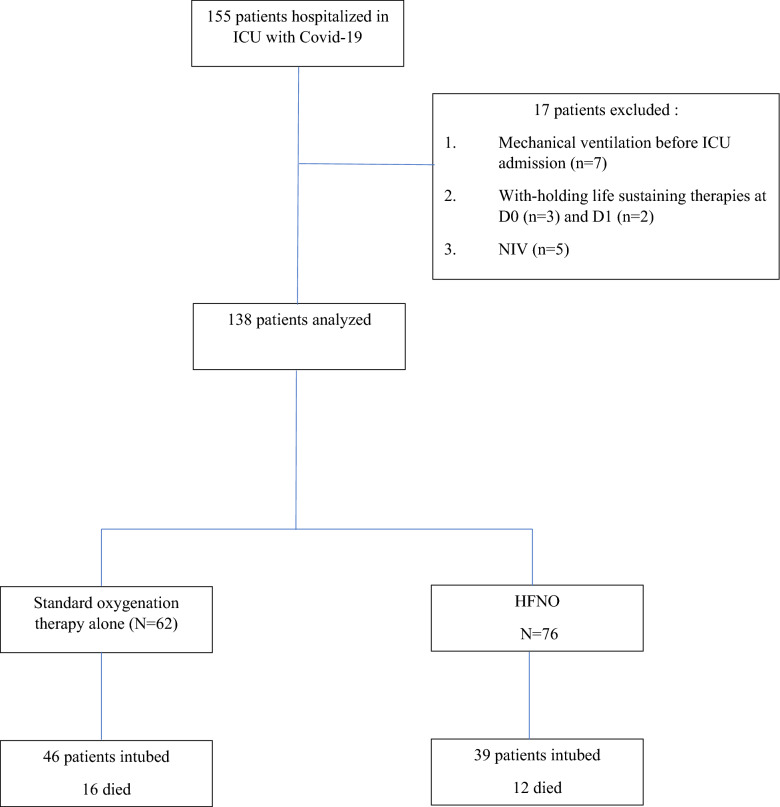
Flow chart. *NIV* non-invasive mechanical ventilation, *ICU* Intensive Care Unit, *HFNO* High Flow Nasal Canula Oxygen

A total of 155 COVID-19 patients were admitted in the participating ICUs for COVID-19. Among them, 17 had non-inclusion criteria (invasive mechanical ventilation before ICU-admission *n* = 7, decision to withdraw life-sustaining therapy *n* = 5, non-invasive mechanical ventilation *n* = 5) (Fig. 1). Among the remaining 138 patients, 62 (45%) were treated with standard-oxygen therapy alone, and 76 (55%) with HFNO. In the vast majority, HFNO was initiated in the 24 h following ICU admission (66/76 patients [89%]).

Characteristics of the patients at ICU admission are presented in Table 1.

**Table 1:** Characteristics of patients at ICU admission*

	Overall (*n* = 138)	Standard oxygen therapy (*n* = 62)	HFNO (*n* = 76)	*p*
Demographic and clinical characteristics				
Male—no. (%)	112 (81%)	50 (81%)	62 (82%)	1.00
Median age, years (IQR)	59 (48–67)	60 (51–67)	60 (52–67)	0.64
Immunocompromized patient—no. (%)	20 (14%)	9 (14%)	11 (14%)	1.00
Diabetes—no. (%)	43 (31%)	19 (31%)	24 (32%)	1.00
Hypertension—no. (%)	56 (41%)	19 (31%)	37 (49%)	0.049
Chronic respiratory failure—no. (%)	4 (3%)	2 (3%)	2 (3%)	1.00
Chronic kidney failure—no. (%)	7 (5%)	3 (5%)	4 (5%)	1.00
Median BMI, kg/m^2^ (IQR)	29 (26–33)	27 (26–33)	29 (25–33)	0.59
Median SAPS II (IQR)	36 (26–46)	35 (26–45)	36 (27–47)	0.62
Median time from onset symptoms to ICU admission, days (IQR)	10 (8–13)	8 (8–13)	10 (8–13)	0.002
Respiratory finding				
Median 02 flow, liter/minute (IQR)	9 (4–12)	6 (5–13)	9 (6–15)	0.003
Median PaO2, mmHg (IQR)	71 (64–84)	71 (63–85)	69 (63–82)	0.45
Median respiratory rate, (IQR)	30 (27–35)	30 (26–35)	33 (28–36)	0.018
Median O2 maximal flow rate, Liter/minute, (IQR)	12 (9–15)	12 (9–15)	12 (9–15)	0.049
Laboratory finding				
Median Lymphocyte, G/L (IQR)	0.8 (0.6–1.1)	0.9 (0.6–1.1)	0.8 (0.6–1.0)	0.10
Median Fibrinogen, g/L (IQR)	6.59 (5.9–7.3)	6.57 (6.0–7.3)	6.7 (6.2–7.3)	0.50
Median Phosphor, mmol/L (IQR)	0.82 (0.69–0.95)	0.8 (0.71–0.94)	0.84 (0.70–0.94)	0.29
Median CRP, mmol/L (IQR)	182 (114–263)	182 (106–262)	182 (125–269)	0.64
Median ferritin, µg/L (IQR)	1502 (874–2530)	1137 (880–2495)	1701 (885–2677)	0.036
Chest CT scan finding				
Median ground glass surface, % (IQR)	50 (25–60)	50 (25–60)	50 (25–60)	0.27
Steroids during ICU stay—no.(%)	66 (48%)	25 (40%)	41 (54%)	0.15

^*^HFNO high flow nasal canula oxygen, *SAPS II* Simplified Acute Physiology Score, *BMI* Body Mass Index, *CRP C*-reactive protein, ICU Intensive Care Unit

### Primary endpoint

**Fig. 2:**
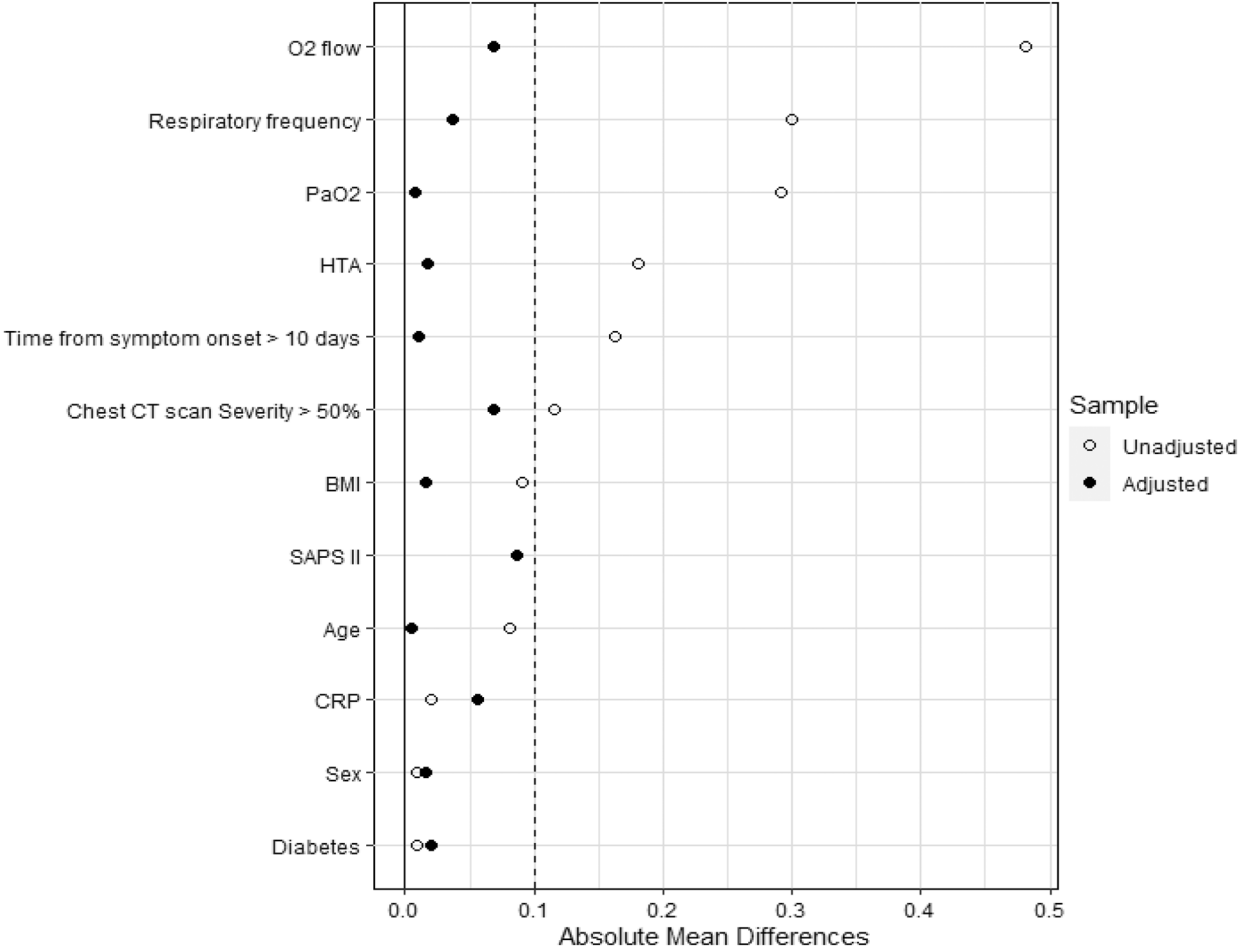
Mean difference of covariate balances before and after weighting

In the standard-oxygen therapy group, 46/62 (74%) patients finally received IMV during the ICU stay compared with 39/76 (51%) in the HFNO group (*p* = 0.007). Patients in the HFNO group were more likely to have hypertension (49% vs 31%, *p* = 0.0049), and had a higher time from the onset of symptoms to ICU admission (10 days vs 8 days, *p* = 0.002). Moreover, patients in HFNO group were more severely ill at the time of ICU admission as attested by a higher flow rate of oxygen before ICU admission (9 l/min vs 6 l/min, *p* = 0.003), and a higher respiratory rate (33 per minute vs 30 per minute, *p* = 0.018). Covariate balances before and after weighting are reported in Fig. 2. HFNO was associated with a significantly lower rate of IMV after standard logistic regression and after weighted propensity score (OR 0.37 [95% CI, 0.18–0.76] p = 0.007 and OR 0.31 [95%CI, 0.14–0.66] *p* = 0.002, respectively) (Table 2). The two sensitivity analyses performed for the primary outcome using a truncated IPTW excluding patients with an extreme IPTW (5th-95th percentile) and excluding HFNO patients with oxygen flow < 50L/min did not change these results (Additional file 2: Table S1).

**Table 2:** Primary and secondary outcomes

Outcomes	Standard oxygen therapy	HFNO	unadjusted		IPTW	
			OR [IC95%]	*p*	OR [IC95%]	*p*
IMV—no (%)	46/62 (74%)	39/76 (51%)	0.37 [0.18–0.76]	0.007	0.31 [0.14–0.66]	0.002
Death at D28—no (%)	15/62 (24%)	9/76 (12%)	0.42 [0.17–1.04]	0.061	0.52 [0.2–1.34]	0.17
Death at D60—no (%)	16/62 (26%)	12/76 (16%)	0.54 [0.23–1.25]	0.15	0.75 [0.32–1.8]	0.52
Ventilator free days, days (IQR)	10 (0–27)	21(5–28)	–	0.005	–	–
Mean length of stay, days (IQR)	12.5 (4–24)	11 (5–20)	−0.04 [−0.35 to 0.27]	0.78	−0.23 [−0.54 to 0.08]	0.14

*IMV* invasive mechanical ventilation, *HFNO* high flow nasal canula, *IPTW* inverse probability of treatment weighting, *IQR* interquartile

### Secondary endpoints

Mortality at day 28 and day 60 did not significantly differ between HFNO group and standard-oxygen group (12% vs 24%; OR 0.52 [95%CI, 0.2–1.34] *p* = 0.17 and 16% vs 26%; OR 0.75 [95%CI, 0.32–1.8] *p* = 0.52, respectively) even after weighted propensity score. The mean length of ICU stay did not differ after weighted propensity score in HFNO group compared to the standard oxygen therapy group (11.0 days vs 12.5 days; difference -0.23 [95%CI, -0.54—-0.08] *p* = 0.14). The number of ventilator-free days at days 28 was higher in HFNO group compared to the standard oxygen therapy group (21 days vs 10 days, *p* = 0.005).

In patients receiving HFNO, a ROX index of higher than 4.88 [17] was associated with a lower risk of IMV (OR 0.23 [95%CI, 0.008–0.64] *p* = 0.006) and a higher SAPS2 score with a higher risk of IMV (OR 1.13 [95%CI, 1.06–1.20] *p* = 0.0002) (Additional file 3: Table S2).

During the first period (before March 27), 5 patients received HFNO. After the decision to not to restrict the use of HFNO (second period), 71 patients received HFNO. Among the 172 health-care workers, 14 (8%) had clinical signs of SARS-CoV-2 infection and 6 (3%) had a positive PCR SARS-CoV-2 during the first period. These figures were 4 (2%) and 0 during the second period.

## Discussion

This retrospective bicenter study shows, in a population of severe COVID-19 patients with ARF, that an initial oxygenation strategy including the use of HFNO is associated with a lower rate of IMV.

In a recent large French observational study (COVID-ICU [18]) of COVID-19 patients hospitalized in ICU, HFNO were applied to 19% of patients compared to 55% in the present study. We have no details about the criteria for IMV in COVID-ICU study, but we suppose that the hypothetical risk of aerosol dispersion led to calls for early intubation [5], leading the clinician to discourage the use of non-invasive modalities including HFNO. Interestingly, the authors of COVID-ICU reported an increase use of HFNO over the study period (from 15% before March 15 to 35% after April 16). This change over time may be explained by the absence of strong argument for the risk of transmission to healthcare workers associated with the use of HFNO.

Until recently, most studies on HFNO and severe COVID-19 were case reports or expert opinion[19]. In October 2020, a retrospective study by Demoule et al. was conducted to assess the efficacy of HFNO in patient with COVID-19 admitted for ARF in ICU [4]. Demographic, clinical, and biological characteristics of these patients were roughly similar to those in our study. Among the 379 patients included, 146 (39%) received HFNO and the use of this technique was associated with reduced proportion of patients requiring IMV (55% vs 72% after adjusting of propensity score, *p* < 0.0001) which is consistent with our findings. However, neither in the study by Demoule et al. nor in our study the use of HFNO affected case fatality. One should note that ICU teams who performed these both studies routinely use HFNO for hypoxemic ARF since many years. This may partly explain the higher proportion of patients who received HFNO compared to the large French observational COVID-ICU study.

In our study, mortality rate at day 60 did not significantly differ between groups. However, this may be explained by a lack of power.

A retrospective study performed in China and published in March 2020 showed that HFNO was the most common ventilator support for SARS-CoV-2 pneumonia [20]. The authors observed that an increasement of respiratory rate and FiO2 in were associated with failure of HFNO which is reliable to our finding. Indeed, in a complementary analysis, we showed that ROX index (which including notably the FiO2 and the respiratory rate and was define as follow SpO2/FiO2/RR) was associated with failure of HFNO. This is consistent with a recent study on prediction of outcome during COVID-19 ARF [21].

In a prospective study, Montiel et al. suggested that a surgical mask placed on patient’s face treated with HFNO improves oxygenation [22]. Our study was not designed to evaluate this effect or a potential association with a lower risk of IMV. However, wearing a face mask was strongly recommended when HFNO was used in all participative centers.

In the general population, several studies have compared HFNO with non-invasive ventilation and standard oxygen. HFNO has been shown to be better tolerated than other non-invasive strategies [23]. It provides better oxygenation compared with standard oxygen [24]. Few randomized clinical trials comparing the clinical efficacy of these various non-invasive approaches have been conducted in the last decade. Frat et al., compared HFNO to standard oxygen using non-rebreather face mask and to non-invasive ventilation. There was no statistical difference in IMV rate between the three groups with 37%, 47% and 50%, respectively [3]. However, in a subgroup analysis of most severe hypoxemic patients (PaO2/FiO2 < 200), the IMV rate was significantly lower in HFNO group.

One of the main issues of the COVID-19 outbreak in France was the management of the capacity of our ICUs. As the COVID-19 pandemic spread, French ICUs were physically and materially challenged by the associated immense caseload. One of the main causes for ICUs being overwhelmed is the high mean length of invasively mechanical ventilation in COVID-19 patient [25]. Our study shows that the need for IMV is reduced by the use of HFNO. This information is of great importance, since all strategies that reduce workload in an ICU should be promoted.

The study has several limitations. First, the analysis of ventilator-free- days should be viewed with caution. Indeed, because of non-normal distribution, ventilator-free days were unadjusted for potential confounders. Second, this is a not a randomized controlled study. So, the results should be interpreted with caution because of potential biases inherent in the non-interventional design. However, to reinforce the strength of our study, we use a propensity score analysis to balance the difference in 12 baseline variables selected before analysis, between the two groups. Finally, our study took place over almost 2 months at different stages of the outbreak, during which the standard of care evolved. To build our propensity score, we did not take into account the potential effect of immunomodulatory treatment.

An Italian observational study suggested that the use of non-invasive respiratory support delivered outside the ICU was associated with risk of staff contamination [26]. We also evaluated the risk of transmission of SARS-CoV-2 to health-care workers when we used HFNO and we observed a low risk of transmission. However, these results should be interpreted with caution. Firstly, we did not take into account the stage of the outbreak. Indeed, SARS-CoV-2 dissemination in French population was probably higher in the first period of the outbreak. This may have influenced the number of new contaminations in our ICU team. Secondly, the heath-care workers may have been more cautious with patients receiving HFNO, because they were aware of a potential higher risk of contamination at the beginning of the outbreak. Thirdly, there was no systematic PCR or serology performed on the totally of the workers. In these conditions, and because our study was not performed to answer this question, it is not possible to confirm any cause-and-effect relationship between oxygen strategy and the risk of transmission of SARS-CoV-2 to health-care worker. However, our data suggest that the risk of SARS-CoV-2 transmission is low and that HFNO could safely be used more widely.

## Conclusion

In this retrospective observational study, the use of HFNO in COVID-19 patient with acute respiratory failure was associated with a lower risk for invasive mechanical ventilation.

## Supplementary Information

**Additional file 1: Figure S1** Plot - missing data for each variables.

**Additional file 2: Table S1.** sensitivity analysis with IPTW truncated at 95% and after excluded HFNO flow rate < 50L/min.

**Additional file 3: Table S2.** Predictive factors associated with invasive mechanical ventilation for patients under High Flow Nasal Oxygenation.

## Data Availability

The datasets used and analysed during the current study are available from the corresponding author on reasonable request.

## Abbreviations

SARS-CoV-2: Severe acute respiratory syndrome coronavirus 2
COVID-19: Coronavirus disease 2019, with acute respiratory distress syndrome
HFNO: High flow nasal canula oxygen
ICU: Intensive care unit
ARF: Acute respiratory failure
CT: Computed tomography
FiO2: Fraction of inspired oxygen
IPTW: Inverse probability of treatment weighting
SOT: Standard oxygen therapy
IMV: Invasive mechanical ventilation
