# Supplementary material for: High flow nasal oxygen therapy to avoid invasive mechanical ventilation in SARS-CoV-2 pneumonia: a retrospective study
Source: Ann Intensive Care. 2021 Feb 27;11:37. doi: 10.1186/s13613-021-00825-5 (PMC7910764; doi:10.1186/s13613-021-00825-5)
Supplement: Supplementary file 1 — Additional file 1: Figure S1 Plot - missing data for each variables. [file 13613_2021_825_MOESM1_ESM.docx]

Additional file 1


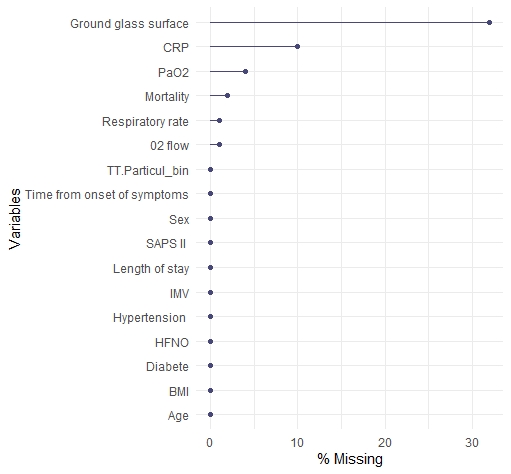


Immunomodulatory treatment

*CRP denotes C-reactive protein, IMV invasive mechanical ventilation, HFNO high flow nasal canula, BMI body mass index, PaO2 Artery partial pressure oxygen, SAPS 2 Simplified Acute Physiology Score

Figure S1 Plot - missing data for each variables*
