# Supplementary material for: High flow nasal oxygen therapy to avoid invasive mechanical ventilation in SARS-CoV-2 pneumonia: a retrospective study
Source: Ann Intensive Care. 2021 Feb 27;11:37. doi: 10.1186/s13613-021-00825-5 (PMC7910764; doi:10.1186/s13613-021-00825-5)
Supplement: Supplementary file 2 — Additional file 2: Table S1. sensitivity analysis with IPTW truncated at 95% and after excluded HFNO flow rate < 50L/min. [file 13613_2021_825_MOESM2_ESM.docx]

Additional file 2: Table S1: sensitivity analysis with IPTW truncated at 95% and after excluded HFNO flow rate < 50L/min*

| Parameters | OR [IC95%] | p |
| --- | --- | --- |
|  |  |  |
| IPTW truncated at 95% | 0.32 [0.15-0.7] | 0.004 |
| IPWT Exclusion of HFNO flow rate < 50L/min | 0.25 [0.11-0.54] | 0.0004 |

*HFNO denotes high flow nasal canula oxygen, IPTW, inverse probability of treatment weighting
