# Supplementary material for: High flow nasal oxygen therapy to avoid invasive mechanical ventilation in SARS-CoV-2 pneumonia: a retrospective study
Source: Ann Intensive Care. 2021 Feb 27;11:37. doi: 10.1186/s13613-021-00825-5 (PMC7910764; doi:10.1186/s13613-021-00825-5)
Supplement: Supplementary file 3 — Additional file 3: Table S2. Predictive factors associated with invasive mechanical ventilation for patients under High Flow Nasal Oxygenation. [file 13613_2021_825_MOESM3_ESM.docx]

Additional file 3: Table S2: Predictive factors associated with invasive mechanical ventilation for patients under High Flow Nasal Oxygenation *

| Parameters | OR [IC95%] | p |  |
| --- | --- | --- | --- |
|  |  |  |  |
|  |  |  |  |
| SAPS 2 | 1.13 [1.06-1.2] | 0.0002 |  |
| ROX index > 4.88† | 0.23[0.08-0.64] | 0.006 |  |
| Sexe (H) | 0.46 [0.14-1.52] | 0.21 |  |
| Age | 1.03 [0.98-1.07] | 0.24 |  |
| HTA | 0.66 [0.27-1.62] | 0.37 |  |
| Diabete | 0.57 [0.21-1.51] | 0.26 |  |
| BMI | 0.98 [0.91-1.06] | 0.67 |  |
| Time from symptoms onset and ICU admission> 10d | 0.59 [0.24-1.48] | 0.27 |  |
| 02 Flow | 1.01 [0.9-1.14] | 0.83 |  |
| PaO2 | 0.98 [0.95-1.01] | 0.24 |  |
| Respiratory rate | 1.00 [0.94-1.06] | 1.00 |  |
| CRP | 1.34 [0.88-2.04] | 0.18 |  |
| CT scan severity, Surface >50% | 0.84 [0.3-2.33] | 0.74 |  |

*HTA denotes hypertension, BMI body mass index, PaO2 Artery partial pressure oxygen, SAPS 2 Simplified Acute Physiology Score, CRP C-reactive protein, CT Computed tomodensitometry

†the latest value of the ROX index within the first 12 h after HFNO initiation[17]
